# A Psychosocial Analysis of the Effect of Body-Contouring Surgery on Patients After Weight Loss

**Published:** 2017-03-28

**Authors:** Khalid J. Alzahrani, Abdullah E. Kattan, Loui A. Ezzat, Saud A. Alsaleh, Khalid A. Murad, Bader A. Alghamdi

**Affiliations:** College of Medicine, King Saud University, Riyadh, Saudi Arabia; and Plastic Surgery Department, King Khalid University Hospital, Riyadh, Saudi Arabia.

**Keywords:** abdominoplasty, body-contouring, psychosocial, truncoplasty, weight loss

## Abstract

**Objectives (Background):** Patients are often bothered by excess skin laxity and redundancy after weight loss. Body-contouring surgery offers a solution. This study assessed the psychosocial impact of body-contouring surgery on patients after weight loss. **Methods (Settings, Design):** In this cross-sectional study, a specifically designed questionnaire developed in collaboration with psychiatric department for our research was used for 43 patients who underwent body-contouring surgery. Data were collected during single visit to the plastic surgery clinic. All the patients had lost 20 kg or less before the surgery and were interviewed at least 6 months after the surgery. The questionnaire was used to compare the psychosocial status of the patients before and after surgery. Data were analyzed appropriately using Statistical Package for the Social Sciences. **Results:** The participants’ mean age was 34 ± 10 years; the sample included 24 (55.8%) women and 19 (44.2%) men (total N = 43). The patients’ quality of life improved significantly in the areas of social life (*P* < .001), job performance (*P* < .002), and sexual activity (*P* < .001). Moreover, while 17 (39.5%) patients suffered symptoms of depression before surgery, only 1 (2.3%) patient suffered symptoms of depression after surgery. The overall satisfaction was found to be 62.8%, with mammoplasty being the procedure with the highest satisfaction (66.6%). **Conclusion:** Body-contouring surgery after weight loss has shown to improve both psychological and social aspects of the patients’ lives. Recall bias is the main limitation in our study.

Obese individuals are at risk of developing numerous health conditions such as diabetes, dyslipidemia, and hypertension, which might cause permanent morbidities and even mortality.^1^^,^^2^ Moreover, the psychosocial image of the obese individuals is negatively affected, causing them to become socially isolated, depressed, and lose confidence in themselves.^3^^,^^4^

Nowadays, awareness of health risks related to obesity is well known, encouraging obese individuals to lose weight and strive toward their ideal bodies.

Losing weight undoubtedly leads to significant health benefits. Obesity-related comorbidities are significantly reduced. Bariatric surgery has a huge impact on morbidity by lowering the risks of diabetes, hypertension, and obstructive sleep apnea. These benefits are noticeable in the majority of patients who undergo bariatric surgery. Remission of type II diabetes mellitus reaches up to 80% 2 years after bariatric surgery.^5^^-^7 Improvement of hypertension by weight reduction is well known, even a modest weight loss can lower blood pressure significantly, hence adding more benefits to surgery to achieve the maximum goal in lowering blood pressure. Finally, the improvement in obstructive sleep apnea was dramatic in almost 80% of patients who underwent surgery.^8^

However, extremely obese patients often struggle to lose a significant enough amount of weight that will reduce morbidity and mortality. Bariatric surgery has been proven to be effective in reducing weight and improving self-image and has become the treatment of choice for many patients.^6^ The number of bariatric procedures is rapidly increasing as evidenced by the statistics. In the United States, 13 365 bariatric surgeries were performed in 1998, 72 177 in 2002, 102 794 in 2003, and 179 000 in 2013.^9^

Although the benefits of bariatric surgery for physical and psychological functions and mental health have been shown,^5^^-^11 many patients complain of excess skin, soft tissue deformity, and hygiene issues. Consequently, the demand for body-contouring surgeries has increased. The physical benefits of these surgeries have been recorded and documented in many studies.^12^^-^14 However, there is little literature published on the psychosocial effects of these procedures. Excess and lax skin after massive weight loss can lead to functional problems and profound dissatisfaction with appearance, resulting in patients to consider body-countering surgeries. Correcting skin excess not only improves body image but also improves the quality of life. In a study by Song et al,^15^ it was noticed that the quality of life and body image improved after body-contouring surgery, while mood remained stable.

This topic still requires a great amount of research to help determine whether these procedures have a positive effect on the patients’ overall mental health, social status, and confidence.

Patients who have lost significant amounts of weight and undergone body-contouring surgery will most likely benefit in psychological and social aspects. We have investigated specific parameters by using questionnaires developed by the in collaboration with the psychiatric department specifically for our study, concentrating on the psychosocial effects of body-contouring surgery. This study evaluated the psychosocial impact of body-contouring surgery on the Saudi population. The results will help the Saudi population in deciding whether body-contouring surgery is a viable option.

## METHODOLOGY

We conducted a cross-sectional observational study on patients who lost at least 20 kg and then underwent body-contouring surgery to remove the ptotic skin that resulted from their weight loss. The study included cross-sectional concurrent and cross-sectional retrospective recall of baseline data. We used a 20-question interviewer-administrated questionnaire to collect the data from our sample. The data were collected from each participant during a single visit to the plastic surgery clinic between December 5, 2013, and March 19, 2014.

This study included patients who underwent body-contouring surgeries in a single center. Moreover, two plastic surgeons (K.A. and B.A.) followed the patients during the period of time reflected in the date.

Our inclusion criteria were that all patients (1) must have had a body mass index between 38 kg/m^2^ and 58 kg/m^2^ before weight loss, (2) must have lost at least 20 kg, and (3) had undergone any type of body-contouring surgery. We excluded patients who had lost weight due to diseases, for example, patients with cancer undergoing chemotherapy, patients who had undergone body-contouring surgery without losing at least 20 kg, and patients who had undergone surgery before 2011. The questionnaire was developed to meet our objectives. Data were collected by interviewing the patients who had follow-up visits in the plastic surgery clinic in the period of our data collection. Our questionnaire was divided into (1) demography, (2) depression and other psychiatric illnesses, (3) the effect of body image, and (4) and rate of satisfaction regarding surgery. We used several variables. Demographics: age, sex, body mass index (kg/m^2^) before surgery, and weight loss method (eg, surgery, pharmacological treatment, or traditional weight loss methods). Psychological: presence of any depressive symptoms before surgery, including but not limited to low mood, anhedonia, and sleep disturbances; whether the patient had visited a psychiatrist before surgery; whether the patient was diagnosed with any psychiatric conditions before surgery; whether the patient was on any psychiatric medications before surgery; whether the patient had any psychiatric conditions at the time of the interview. Social: effect of body image (before and after surgery) on social life, daily activity, work function, and sexual activity; whether the surgery had reached the patient's expectation or not; whether the patient considers the surgery as a good experience or not; whether the patient likes his/her body image after surgery or not; whether there was anything in the surgery that bothered the patient; whether the way people look at the patient changed after the surgery; whether the patient is willing to repeat the surgery, if needed; and the overall satisfaction about the surgery.

We calculated the sample size by choosing the body mass index of the patients before surgery as a single mean. We used the following standard formula for calculating sample size for a single mean:


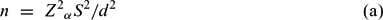


where *S* is the standard deviation, which has been estimated from a similar study found in the literature^16^ (*S* = 10); *D* is the accuracy of estimate, which also has been estimated from the literature considering the feasibility of our research (*d* = 3); and *Z*_a/2_ is the normal deviate that reflects the type I error. Furthermore, we used a 95% confidence interval.

To reduce any selection bias, we used a systematic random sampling technique; using Microsoft Office Excel 2010, we randomly selected a number. We then selected every third patient from our list, which was the follow-up list of patients who underwent body-contouring surgery in King Khalid University Hospital. As calculated using the formula, our mean value was 43 [N = (1.96)^2^ (10)^2^/3^2^ = 43]. However, we included 60 patients instead of 43 in case we needed to exclude participants or if any of the participants refused to fill in the questionnaire.

## STATISTICAL ANALYSIS

For the statistical analysis, we used the Statistical Package for the Social Sciences, version 21. First, we asked the patients about certain variables, for example, how did their self-image affect their sexual life before and after the body-contouring surgery. Second, we analyzed their answers using Statistical Package for the Social Sciences. McNemar's test was used for paired nominal variables; the patients had the option to choose between negatively affected and positively affected. Pearson χ^2^ test was used for categorical variables.

## ETHICAL CONCERNS

The participants were asked whether they were willing to participate in the study and informed of the right to withdraw at any time. Participants who agreed signed a written consent.

## RESULTS

A total of 48 patients were interviewed, 5 were excluded as 3 patients had not lost enough weight (<20 kg) and 2 had undergone surgeries before 2011. In addition, we were unable to interview 9 patients as 4 patients refused to be interviewed due to personal reasons and 5 missed their appointment in the clinic. The response rate was 75%.

The sample descriptors were female (n = 24, 55.8%), male (n = 9, 44.2%). The participant mean was 34 (± 10 years; range: 20-56 years) and mean body mass index was 28 (±3.3 kg; range: 21.60-37.75 kg).

The participants had undergone a variety of body-contouring surgeries ranging from 1 to 3 procedures per participant. Out of the 43 participants, 15 (34%) had undergone mammoplasty alone. Of the participants who underwent multiple surgeries, 5 (11.6%) underwent mammoplasty as one of them, resulting in 20 (46.5%) participants having undergone mammoplasty. The rest of the results are shown in Table 1.

Participants lost weight using 3 different methods; the majority of the patients (30, 69.8%) lost weight by using a nonsurgical method such as diet and exercise. Furthermore, 12 (27.9%) participants lost weight through bariatric surgery, and only 1 (2.3%) participant lost weight by using medications. All of these results are presented in Table 2. A total of 12 (27.9%) participants had undergone bariatric surgery as a method of weight loss. Of these patients, 6 (13.9%) underwent sleeve gastrectomy, 3 patients (7.0%) underwent banding, and 3 (7.0%) underwent gastric bypass. All these results are presented in Table 3.

When asked about their opinions on the surgery, 37 participants were pleased with the results of their surgery, 1 participant was not sure, and only 5 were displeased or dissatisfied. Moreover, 27 participants reported that the surgery had met their expectations, 7 participants were not sure, and 7 others reported that the surgery did not meet their expectations. Furthermore, self-image after the surgery was satisfying according to 24 participants, equivocal according to 9 participants, and not satisfying according to 10 participants. However, no statistical significance was found when comparing different body-contouring surgeries to patient's opinion (*P* = .613), patient's expectations (*P* = .747), and self-satisfaction (*P* = .405). These results are shown in Table 4.

Furthermore, when patients were asked about the public's view on their body appearance, 24 participants reported an improvement, 11 reported no change, 8 were not sure, and no participants reported worsening of their appearance after surgery. When asked whether or not they would repeat their surgery if they had the chance, 35 participants would repeat it, 5 were not sure, and 3 would not repeat their surgery. In addition, complete satisfaction with the surgery was reported by 27 (62.8%) participants, partial satisfaction was reported by 13 (30.2%) participants, and only 3 participants (6.98%) were dissatisfied. Patients who underwent mammoplasty particularly had a higher percentage of satisfaction (66.6%). However, there was no significant statistical difference when comparing different body-contouring surgeries to participant satisfaction (*P* = .216), likelihood of repeating the surgery (*P* = .381), and public view of appearance (*P* = .518). These results are shown in Table 5.

A total of 17 (39.5%) participants had reported symptoms of depression before undergoing the body-contouring surgery, but only 2 (4.7%) participants consulted a psychiatrist. However, only 1 (2.3%) participant felt depressed after surgery. As shown in Table 6, there is a statistically significant difference (*P* < .001) when comparing the number of reported depression cases before and after surgery.

A total of 37 (86%) participants felt that their self-image prior to the body-contouring surgery negatively affected their social life, while only 6 (14%) participants reported not being negatively affected. However, 36 (83.7%) participants felt that their self-image after body-contouring surgery had positively affected their social life, while only 7 (16.3%) patients complained of it negatively affecting their social life. As shown in Table 6, the results evidence a statistically significant difference (*P* < .001) between the variables corresponding to the effect on social life before and after surgery.

A total of 21 (48.8%) participants complained of their self-image hindering their daily activities, and 22 (51.2%) participants stated that their daily activities had not been negatively affected by their self-image. On the contrary, 41 (95.3%) participants reported a positive effect on their daily activities after body-contouring surgery, leaving only 2 (4.7%) participants claiming no positive effect of their body-contouring surgery on their daily activities. Furthermore, as seen in Table 6, when comparing the 2-paired variables, a statistically significant result (*P* < .001) was found.

A total of 11 (25.6%) participants reported that their self-image was negatively affecting their job performance, and 32 (74.4%) of the participants stated that they had not been negatively affected. However, 42 (97.7%) participants reported positive results when asked about their self-image affecting their job performance after body-contouring surgery, while only 1 (2.3%) participant felt negatively affected. After comparing the 2 paired variables, a statistically significant result was found (*P* < .002), as shown in Table 6.

Of the 43 participants, only 24 were married. Furthermore, 12 (50%) participants complained that their self-image was negatively affecting their sex life, and 12 (50%) participants stated that their sex life was unaffected. However, after undergoing body-contouring surgery, 23 (95.8%) participants reported an improvement in their sex life, and only 1 (4.2%) participant reported no effect. After comparing the paired variables, the results were shown to be statistically significant (*P* < .001), as shown in Table 6.

## DISCUSSION

Although bariatric surgery has been proven to be a considerable solution for those who need to lose weight,^17^^,^^18^ patients are usually dissatisfied because of the excess skin.^16^ Hence, patients are choosing body-contouring surgery as a solution to this problem. However, only a few studies have been carried out to study the psychosocial aspects of patients’ lives after these surgeries.^19^

When comparing our findings with the results of other studies relevant to ours,^19^ we identified that they are consistent regarding the social aspects. Furthermore, a study from 2011 conducted by Bracaglia et al^20^ showed that body-contouring surgery has increased satisfaction with body experience and image, which is also consistent with our results.

Both the psychological and social improvements have been proved statistically significant when comparing patients before and after body-contouring surgery (*P* < .001 and *P* < .001, respectively). A study conducted in 2012 by Klassen et al^21^ has shown similar improvements in both the psychological and social aspects.

Singh et al^22^ argued that the quality of life of patients who have undergone body-contouring surgery is significantly improved compared with the quality of life before bariatric surgery; however, no significant results were seen when comparing the quality of life in patients who underwent bariatric surgery before and after body-contouring surgery. However, our study showed significant improvements in the daily activity, job performance, and sex life of participants when comparing the data of patients before and after body-contouring surgery. This lack of consistency could be attributed to the fact that some of the patients in our study lost weight by methods other than bariatric surgery.

Azin et al^23^ argued that although body-contouring surgery may benefit the physical quality of life, it does not improve the mental quality of life. Nonetheless, they have found that body-contouring surgery decreases symptoms of depression and anxiety.

All the patients participating in our study were from one single hospital. Furthermore, we used a random sampling technique that allowed us to generalize the results we obtained to similar patients throughout Saudi Arabia and hopefully worldwide.

Our research study has some limitations and could, therefore, be improved. First, our study is liable to recall bias because some of the patients had undergone body-contouring surgery a long time prior to the study. Furthermore, our study included cross-sectional concurrent and cross-sectional retrospective recall of baseline data, which also increases the likelihood of recall bias. Patient's recall may also be influenced by the mood of the patient at the time of the interview.

The strengths of our study are the fact that a questionnaire was designed specifically in cooperation with the psychiatric department for our study, based on our objectives and statistical needs. Furthermore, we targeted patients with 7 different surgical procedures instead of focusing on a single type of body-contouring surgery.

## CONCLUSION AND RECOMMENDATIONS

In conclusion, we have used a self-report questionnaire to assess the psychosocial aspect of patients who have undergone body-contouring surgery after weight loss in a single visit to our plastic surgery clinic. Our results have shown an improvement in the psychological and social aspects in the lives of patients who have undergone body-contouring surgery after weight loss. It has also shown that quality of life in patients after body-contouring surgery has improved in social life, sexual activity, and work domains. Moreover, our results show that a high percentage of patients were satisfied with the results of their operation. Klassen et al^21^ had similar findings, showing an improvement in the psychological and social domains in patients post–body-contouring surgery. Although Azin et al^23^ showed a decrease in symptoms of depression and anxiety, they have found that body-contouring surgery does not actually improve the mental quality of life. The majority of studies in the literature support our findings, deeming our results reliable. As such, bariatric surgery, followed by body-contouring surgery should be considered as the standard management in patients who are morbidly obese who have significant pannus following weight loss greater than 20 kg through any of the procedures explained herein (see Table 2).

To achieve better results, we recommend conducting a prospective cohort study on a large sample of patients who have not yet undergone body-contouring surgery and comparing psychosocial aspects of these patients before and after surgery. We also believe that recall bias has the potential to affect the validity of our results, particularly because data were collected in a single visit.

## Figures and Tables

**Table 1:** The various types of body-contouring surgeries undertaken by the participants*

Type of body-contouring surgery	Frequency	%	Cumulative %
Abdominoplasty	10	23.3	23.3
Abdominoplasty, brachioplasty, and thigh lift	1	2.3	25.6
Brachioplasty	2	4.7	30.2
Mammoplasty	15	34.9	65.1
Mammoplasty and abdominoplasty	2	4.7	69.8
Mammoplasty and brachioplasty	3	7.0	76.7
Neck liposuction	1	2.3	79.1
Thigh lift	3	7.0	86.0
Truncoplasty	6	14.0	100.0
Total	43	100.0	

*Results are expressed as frequency, percent, and cumulative percent (discrete variable).

**Table 2:** Various methods of weight loss (discrete variable)

Weight loss method	Frequency	%	Cumulative %
Diet and exercise	30	69.8	69.8
Medications	1	2.3	72.1
Bariatric surgery	12	27.9	100.0
Total	43	100.0	

**Table 3:** Various types of bariatric surgery that were used by the patients in this study (discrete variable)

Type of bariatric surgery	Frequency	%	Cumulative %
Banding	3	7.0	7.0
Gastric bypass	3	7.0	14.0
Sleeve gastrectomy	6	13.9	27.9
NA	31	72.1	100.0
Total	43	100.0	

**Table 4:** Comparisons of the type of body-contouring surgery undergone by the participants with (1) patient's expectations, (2) patient's opinion of surgery, and (3) patient's satisfaction of self-image after surgery (categorical variable)

		Type of body-contouring surgery									
		Truncoplasty	Abdominoplasty	Mastoplasty	Brachioplasty	Thigh lift	Liposuction	Multiple surgeries	Total	Value	Pearson χ^2^ symptom Significance. (2 sided)
Did surgery reach your expectation	Yes	5	6	10	1	2	0	3	27	6.774	0.747
	No	1	2	1	1	1	0	1	7		
	Maybe	0	3	4	0	0	0	2	7		
Opinion of surgery	Good	5	11	13	1	2	0	5	37	8.166	0.613
	Bad	1	0	1	1	1	0	1	5		
	Not sure	0	0	1	0	0	0	0	1		
Satisfaction of self-image after surgery	Satisfied	5	5	9	0	2	0	3	24	10.409	0.405
	Not Satisfied	0	5	2	1	1	0	1	10		
	Not sure	1	1	4	1	0	0	2	9		

**Table 5:** Comparisons of the type of body-contouring surgery undergone by the participants with the (1) public's view of patient's appearance after surgery, (2) likelihood of repeating the surgery, and (3) degree of patient's satisfaction of the surgery (categorical variable)

	Type of body-contouring surgery										
		Truncoplasty	Abdominoplasty	Mastoplasty	Brachioplasty	Thigh lift	Liposuction	Multiple surgeries	Total	Value	Pearson χ^2^ symptom Significance (2 sided)
Public view of appearance after surgery	Better	5	4	7	2	2	0	4	24	9.154	0.518
	Worse	0	0	0	0	0	0	0	0		
	No change	1	3	4	0	1	0	2	11		
	Not sure	0	4	4	0	0	0	0	8		
Would you repeat the surgery if you had the chance	Yes	5	7	13	2	2	0	6	35	10.7	0.381
	No	1	1	0	0	1	0	0	3		
	Maybe	0	3	2	0	0	0	0	5		
Degree of satisfaction of surgery	Satisfied	5	5	10	1	2	0	4	27	13.138	0.216
	Partially satisfied	0	6	5	1	0	0	1	13		
	Unsatisfied	1	0	0	0	1	0	1	3		

**Table 6:** The effect of body-contouring surgery on patient's (1) social life, (2) marital life (sex), (3) daily activities, (4) job performance, and (5) depression (dichotomous variable)

	Before		After		
	Negative effect	No effect	Negative effect	Positive effect	McNemar's test, *P*
Social	37	6	7	36	*P* < .001
Sex	12	12	1	23	*P* < .001
Daily activities	21	22	7	41	*P* < .001
Job performance	11	32	1	42	*P* < .001
Depression	17	26	1	42	P < 0.001
